# Endosomal Maturation, Rab7 GTPase and Phosphoinositides in African Swine Fever Virus Entry

**DOI:** 10.1371/journal.pone.0048853

**Published:** 2012-11-01

**Authors:** Miguel A. Cuesta-Geijo, Inmaculada Galindo, Bruno Hernáez, Jose Ignacio Quetglas, Inmaculada Dalmau-Mena, Covadonga Alonso

**Affiliations:** Departamento de Biotecnologia, Instituto Nacional de Investigacion y Tecnologia Agraria y Alimentaria (INIA), Madrid, Spain; National Jewish Health, United States of America

## Abstract

Here we analyzed the dependence of African swine fever virus (ASFV) infection on the integrity of the endosomal pathway. Using confocal immunofluorescence with antibodies against viral capsid proteins, we found colocalization of incoming viral particles with early endosomes (EE) during the first minutes of infection. Conversely, viral capsid protein was not detected in acidic late endosomal compartments, multivesicular bodies (MVBs), late endosomes (LEs) or lysosomes (LY). Using an antibody against a viral inner core protein, we found colocalization of viral cores with late compartments from 30 to 60 minutes postinfection. The absence of capsid protein staining in LEs and LYs suggested that virus desencapsidation would take place at the acid pH of these organelles. In fact, inhibitors of intraluminal acidification of endosomes caused retention of viral capsid staining virions in Rab7 expressing endosomes and more importantly, severely impaired subsequent viral protein production. Endosomal acidification in the first hour after virus entry was essential for successful infection but not thereafter. In addition, altering the balance of phosphoinositides (PIs) which are responsible of the maintenance of the endocytic pathway impaired ASFV infection. Early infection steps were dependent on the production of phosphatidylinositol 3-phosphate (PtdIns3P) which is involved in EE maturation and multivesicular body (MVB) biogenesis and on the interconversion of PtdIns3P to phosphatidylinositol 3, 5-biphosphate (PtdIns(3,5)P_2_). Likewise, GTPase Rab7 activity should remain intact, as well as processes related to LE compartment physiology, which are crucial during early infection. Our data demonstrate that the EE and LE compartments and the integrity of the endosomal maturation pathway orchestrated by Rab proteins and PIs play a central role during early stages of ASFV infection.

## Introduction

Many animal viruses have evolved to exploit endocytosis to enter host cells after initial attachment of virions to specific cell surface receptors. African swine fever virus (ASFV), the only known member of the *Asfarviridae* family, is a nucleo-cytoplasmic double-stranded DNA enveloped virus [1]. ASFV particles, with an overall icosahedral shape and an average diameter of 200 nm, are composed of several concentric domains: an internal core consisting of a central DNA-containing nucleoid coated by a thick protein layer referred to as core shell, an inner lipid envelope, and an icosahedral protein capsid [2], [3], [4]. The extracellular virions usually contain an additional external membrane acquired by budding from the plasma membrane [5] and both intracellular and extracellular mature virions are infectious [6], [7]. The viral capsid that surrounds the internal membrane is composed by the major viral capsid protein p72 and protein pE120R [1]. The core shell protein composition consists in a 220 kDa protein that is cleaved to give four structural proteins (p150, p37, p14 and p34) and the two products of a 62 kDa protein [8]. Also, two DNA binding proteins, pA78R and p10 are found in virions. Early mRNA synthesis begins in the cytoplasm immediately after virus entry and is regulated by enzymes and factors packaged in the virus core. Virus DNA replication starts at 6 hours postinfection (hpi) and assembly takes place in perinuclear factory areas [1]. Early genes are expressed prior to DNA replication but some early genes continue to be synthesized throughout infection (e.g. p30 protein encoding gene). The expression of late genes takes place after viral DNA replication. Several structural proteins accumulate in viral factories where virus morphogenesis takes place (p.e. structural proteins p54, major capsid protein p72, etc.) [6], [7]. Most of these studies on viral cycle characterization were performed in the Vero cell line infected with the cell culture adapted isolate ASFV BA71.

Using this model, early studies on ASFV entry demonstrated that the internalization of viral particles is a temperature-, energy-, and low pH-dependent process, since it is inhibited at 4°C and in the presence of inhibitors of cellular respiration or lysosomotropic agents [9], [10]. More recent analysis of major endocytic routes for cell entry indicate that the ASFV moves into Vero cells by clathrin-mediated endocytosis, which requires the activity of the GTPase dynamin [11]. All these features are consistent with a receptor-mediated endocytosis mechanism of entry. Also, the presence of cholesterol in cellular membranes, but not lipid rafts or caveolae, was found to be essential for productive ASFV infection during initial stages. Alternative pathways of entry, such as macropinocytosis have been proposed for cells of the monocyte/macrophage lineage [12] however, these studies encounter the problem that these cells have a heterogeneous surface marker profile and only restricted macrophage subpopulations are susceptible to ASFV [13], [14], [15]. Given that macropinocytosis would also drive to the endocytic pathway at some stage [16], we focused this work on further steps in endocytosis that remain unexplored.

Once a virus has entered the endocytic pathway, it must temporally and physiologically pass through distinct endosome populations to achieve successful infection; however, it is still unknown whether ASFV follows this pathway. Early endosomes (EEs) generally serve as sorting vesicles for incoming ligands, such as epidermal growth factor (EGF) or transferrin. The mode of entry of these ligands by clathrin-mediated endocytosis is very fast and efficient and recruitment of the necessary molecules to clathrin vesicle scission have been determined to occur within a 30–100 seconds (s) time frame [17], [18]. EEs can progress to recycling endosomes (REs), which deliver endocytosed material back to the cell surface, or progress and mature to late endosomes (LEs). LEs have a significantly lower pH compared to that of EEs [19] and may fuse with lysosomes (LYs) for degradation. Multivesicular bodies (MVBs) form through invagination of small intraluminal vesicles (ILVs) and thus EE become MVBs. The development of these vessels requires the endosome-specific lipid phosphatidylinositol 3-phosphate (PtdIns3P) [20]. As EEs undergo maturation, the lumen (pH of 6.5) is gradually acidified to reach a pH of 6–5 in MVBs and mature to Rab7-expressing LEs. After the fusion of LEs with LYs, which are characterized by Lamp1 expression, a pH of 5–4.5 is reached.

Endosome maturation requires a coordinated function of lipids and proteins in membrane bending, elongation and fission processes. These cellular factors are hallmarks of the steps followed by viruses to traffic through the cytoplasm. Members of the Rab family of small GTPases are regulators of the host endocytic pathway and each Rab member localizes to a specific compartment [21]. By interacting with one or more effector proteins, Rab proteins create membrane subdomains to regulate specific downstream functions, such as membrane transport and fusion by recruiting tethering and docking factors [22]. Rab5 regulates fusion between EEs and the motility of these compartments along microtubules. In contrast, Rab7 acts more downstream in the endocytic pathway and, it controls transport to LEs and regulates the transport of and fusion between LEs and LYs [23].

Regulation of the endosomal pathway by Rab GTPases is achieved in coordination with the lipid composition of the endosomal membrane in phosphoinositides (PIs). This regulates the traffic and maturation of the endosome since these molecules allow the specific incorporation of binding proteins to a given membrane. PIs, which are the phosphorylated forms of phosphatidylinositol (PtdIns) are tightly regulated both spatially and temporally through the many phosphoinositide kinases (PIKs) and phosphatases by rapid metabolic interconversions. The regulatory actions of PIs in many cellular functions are the result of their capacity to control the subcellular localization and activation of various effector proteins that carry PI-binding domains, such as the PH (Pleckstrin homology), FYVE (Fab1p, YOTB, Vac1 and EEA1), and PX (Phox homology) domains [24], [25]. All these cellular factors may be required for virus traffic through the endocytic pathway.

Viruses have evolved to exploit the endocytic pathway for cell entry and transport. Members of subgroup B of Adenovirus (Ad), serotypes 3 and 7, have relatively long residence times inside endosomes. The endosomal pathway was identified as the route used by Ad7, as virions were observed to colocalize with LE and LY marker proteins, including Rab7 and Lamp1, during viral entry and before viral egress from this compartment [26]. Despite trafficking through this pathway, Ad7 escapes degradation in these organelles. This virus traffics through the low lysosomal pH and the Ad fiber protein confers the property of low pH escape of the Ad7 capsid to the cytoplasm. Colocalization studies of the influenza virus using defined endosomal markers and Rab mutants, confirmed the traffic of this virus through EEs and LEs at distinct times during infection. The HA glycoprotein of the influenza virus has been described to undergo membrane fusion at a pH of 5.5. Analysis with conformation-specific antibodies against HA indicates that the fusion peptide is not exposed until late in entry, approximately at the time when virus is concentrated in LEs [27].

Here we studied the endocytosis of ASFV as it traffics through the cytoplasm during early infection. ASFV required functional EEs and LEs during early steps for successful infection, together with Rab5 and Rab7 GTPases – master regulators of the endocytic pathway – and membrane PI signaling. We analyzed the temporal passage of the virus through the different endosomal compartments and studied the impact of endosome maturation processes on ASFV entry into the host cell.

## Results

### Relevance of endosomal compartments during ASFV infection

To study how ASFV gains access to the host cell through the endocytic pathway, we examined the virus association with early and late endosomal compartments as it traffics to reach its replication site at the microtubule organizing center (MTOC). The colocalization of ASFV proteins detected in virions such as p72 major capsid protein and pE120R with endosome markers was analyzed by confocal immunomicroscopy of fixed cells.

**Figure 1:**
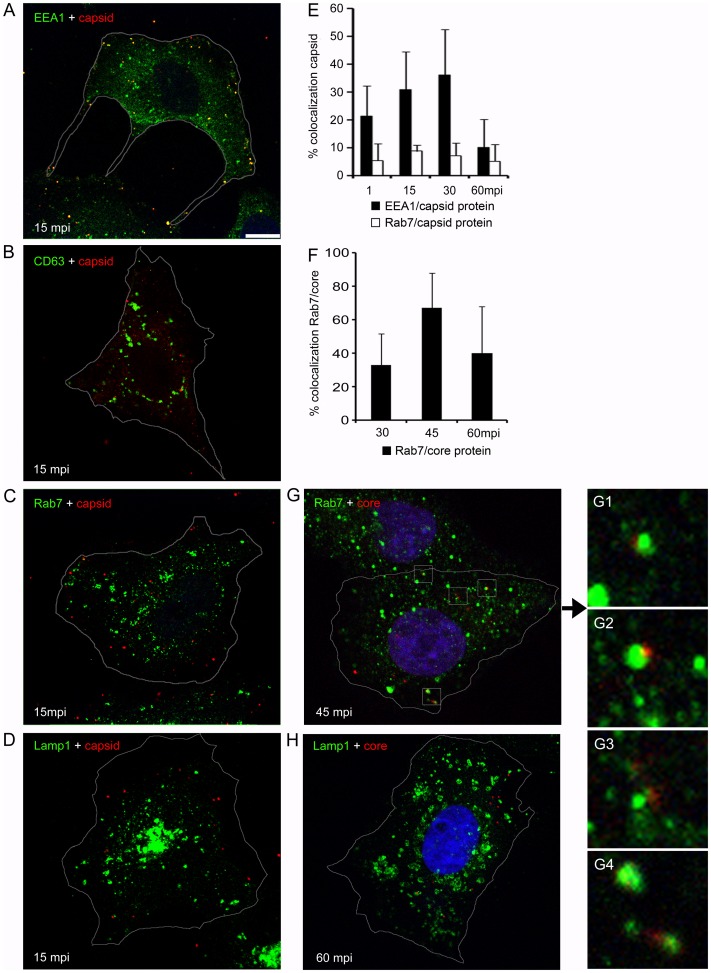
Viral colocalization with endosomes at early infection. Representative confocal micrographs of Vero cells infected with ASFV and immunostained for viral capsid proteins p72 and pE120R (shown in red), and in green, EE marker EEA1 (**A**), MVB marker CD63 (**B**), LE marker Rab7 (**C**) and LY marker Lamp1 (**D**) at 15 minutes postinfection (mpi). Scale bars, 10 µm. Cells were infected at a moi of 10 pfu/cell and adsorption was maintained at 4°C for 90 min. Unbound virus was then washed, cells were shifted to 37°C and infection was allowed to progress for indicated times. (**E**) Percentages of colocalization events of p72 capsid protein with EE or LE marker are expressed as means and relativized to the total cell-associated virus particles per individual cell at each time point in 10 cells in duplicates. (**F**) Percentages of colocalization events of p150 inner core protein with LE marker expressed as means and relativized to the total cell-associated virus particles per individual cell at each time point in 10 cells in duplicates. (**G**) Representative confocal micrograph of the colocalization of viral cores with Rab7 positive endosomes. Nuclei were stained with TOPRO3. (**G1–4**) Detail of colocalization between viral cores and LE in high magnification of the boxed areas in (G). (**H**) Colocalization of viral core protein p150 with Lamp1 marker.

Vero cells were infected with ASFV BA71V isolate at a multiplicity of infection (moi) of 10 pfu/cell. This experiment was performed at a high moi in order to visualize several viral particles per cell. After virus adsorption at 4°C for 90 min, medium was replaced by warm medium at 37°C, and cells were then fixed at the indicated time points starting from 1 min post-infection (mpi) to 60 mpi. To detect EEs, MVBs, LEs and LYs, we used antibodies against EEA1, CD63, Rab7 and Lamp1, respectively (Fig. 1 left panel). Confocal microscopy analysis of cells infected with ASFV showed increasing colocalization of viral capsid protein with EEA1 from 1 to 30 mpi, to decrease thereafter (Fig. 1 A, E). In contrast, p72/pE120R viral capsid proteins showed a very low colocalization level with CD63, Rab7 and Lamp1 (Fig. 1 B–E). The absence of capsid protein staining in late endosomes and lysosomes suggested that desencapsidation of virions could occur at the acid pH of these compartments. Then, we labeled virions with an antibody against p150 viral core protein. Staining for inner viral core protein p150 corresponding to desencapsidated virions, was found in over 60% Rab7 positive endosomes at 45 min after infection (Fig. 1 F, G and G1–4). In contrast, viral core protein colocalization was reduced to ca. 15% with Lamp1 between 30–60 mpi (Fig. 1 H). This fact, could suggest that the virus uncoating and egress to the cytosol occurred rapidly from LEs and few viral cores colocalized with lysosomes expressing Lamp1.

### Low intraluminal pH and Endocytosis are required for ASFV infectivity

**Figure 2:**
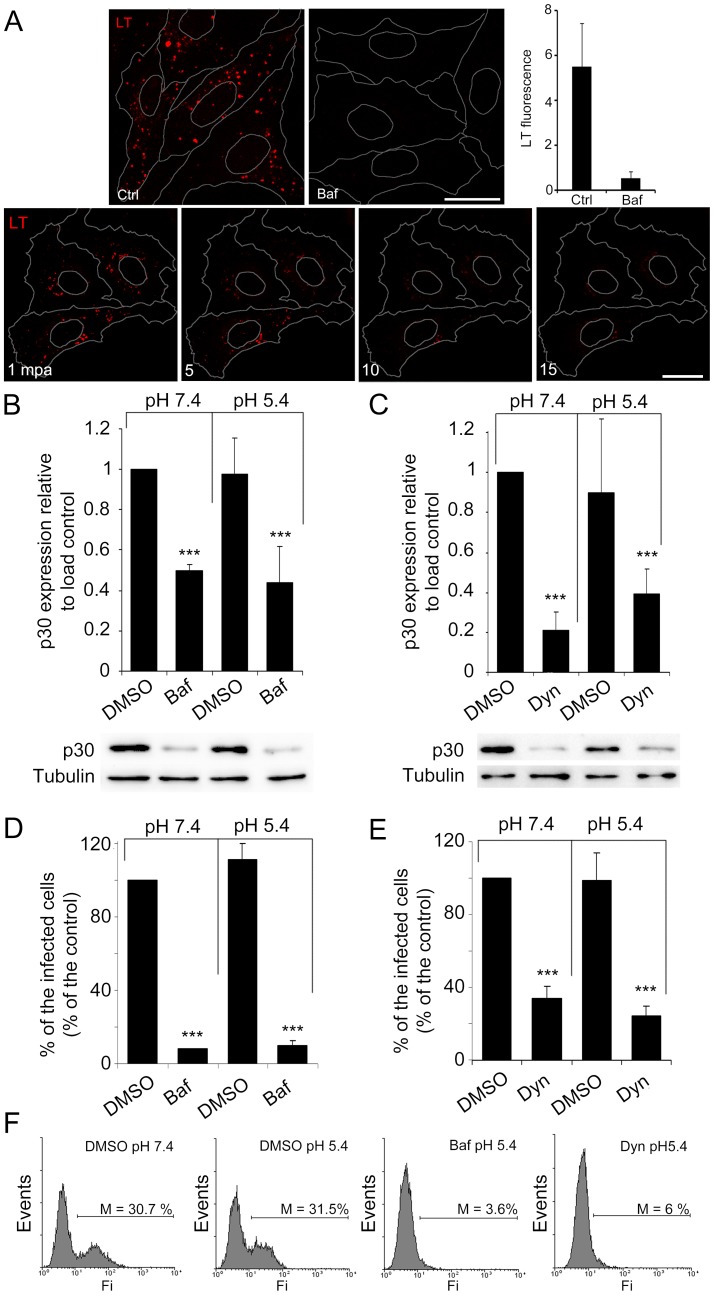
Low intraluminal pH and Endocytosis are required for ASFV infectivity. (**A**) Inhibition of intraluminal acidification of endosomes by Baf in a time dependent manner is shown using the pH sensitive dye lysotracker red. Bar 25 µm; mpa: minutes after Baf addition. (**B–E**) Inhibition of intraluminal acidification of endosomes with Baf and inhibition of endocytosis with Dyn impaired virus infectivity and neither Baf nor Dyn inhibition of early infection could be recovered with acid pH medium. (**B**) Early viral protein p30 expression in cells pretreated with 200 nM Baf or DMSO and pulsed for 1 h with ph 5.4 medium postadsorption or maintained at pH 7.4 for 6 hpi. Western blot with specific antibodies was quantified and normalized to protein load control values. Low early viral protein expression with Baf was not recovered by acid pH medium treatment. (**C**) Quantification of viral protein p30 expression at 6 hpi as determined by Western blot in cells pretreated with 80 μM Dyn or DMSO and maintained in presence of medium at pH 7.4 or pulsed at pH 5.4 for 1 h post-adsorption. (**D**) Flow cytometry of Vero cells pretreated with Baf and infected in medium at pH 7.4 or pulsed at pH 5.4 for 1 h post-adsorption. Infected cells were then detected by FACS and data normalized to infection rates in DMSO treated cells. (**E**) Flow cytometry of Vero cells pretreated with Dyn and infected in medium at pH 7.4 or pulsed at pH 5.4 for 1 h. Asterisks denote sadistically significant differences (*** *P<0.001*). (**F**) Representative FACS profiles obtained during the analysis are shown.

To study the possibility that the virus desencapsidation occurred at the acid pH of LEs, we analyzed the effects of inhibitors known to raise the luminal pH of endosomes. As EEs undergo maturation, the lumen (pH of 6.5) is gradually acidified to reach a pH of 6–5 in MVBs and mature to Rab7-expressing LEs. After the fusion of LEs with LYs, which are characterized by Lamp1 expression, a pH of 5–4.5. The acidic pH of the late compartment stages of the endosomal pathway in contrast to EEs was shown in Vero cells using a pH sensitive dye (lysotracker; Fig. S1 A–D). The vacuolar ATPase (V-ATPase) is required for acidifying endosomes and LYs [28]. Treatment of Vero cells with V-ATPase inhibitor bafilomycin A1 (Baf) caused a rapid alkalinization of these organelles as monitored with the pH sensitive dye which resulted in a rapid reduction in lysotracker fluorescence (Fig. 2 A).

The inhibition of acidification would thus impair viral particles uncoating in LEs and could eventually stop further progress of viral infection. Nevertheless, the presence of viral particles in the cytoplasm could not be representative of their capacity to pursue a successful infection. Hence, in order to evaluate the actual impact on a productive infection we addressed the following infection step corresponding to early viral protein synthesis.

We treated cells with 200 nM Baf to inhibit endosome acidification. Cells were infected at 1 pfu/cell and after a brief adsorption period (90 min) at 4°C, medium was replaced and temperature was shifted to 37°C (time 0). Thereafter, a pulse of acid pH medium at 5.4 for 1 h was performed when indicated, followed by washing. Infection was then allowed to proceed for 6 hpi with medium at pH 7.4. Then, acid pH pulsed, Baf treated and control cells were collected and early protein p30 expression was evaluated by Western blot and flow cytometry. P30 is a viral protein that is expressed early post infection and during the complete viral cycle. Under Baf treatment, early protein expression decreased indicating the relevance of the intraluminal low pH and this effect could not be reversed by weak acid treatment of the cells (Fig. 2 B, D).

Conversely, we assayed whether acid pH could allow infection in absence of endocytosis. ASFV was allowed to adsorb to Vero cells in presence of dynamin inhibitor dynasore (Dyn) at 80 µM concentration and then medium was replaced with medium at pH 5.4 (1 h acid pH pulse) or pH 7.4 and dimethyl sulfoxide (DMSO) control. After 6 hpi, cells were collected and early protein p30 expression evaluated by western blot and flow cytometry (Fig. 2 C and E, respectively). Acid media conditions did not allow infectivity recovery in the presence of endocytosis inhibitor Dyn.

### Time-dependent effect of lysosomotropic drugs on ASFV infectivity

**Figure 3:**
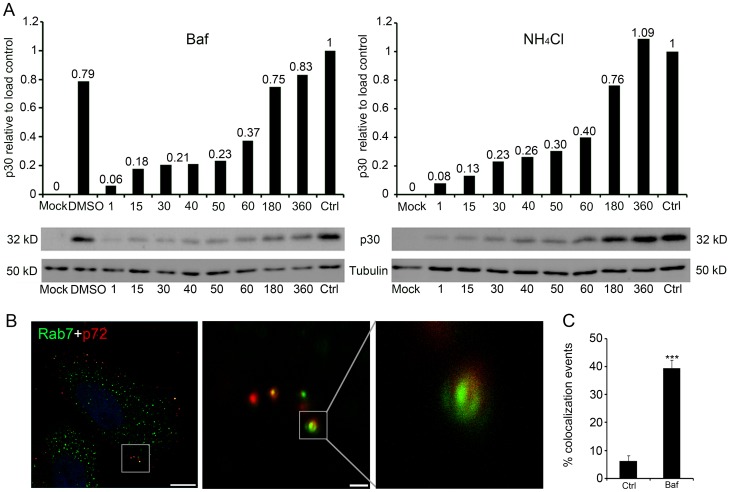
Acid pH of the late endosome is required at early stages of ASFV infection. (**A**) Early viral protein p30 expression determined at 8 hpi by Western blotting with specific antibodies, quantified and normalized to protein load control values. Acid pH requirement was evidenced by the effect of lysosomotropic drug addition at any time point within the first hpi but not thereafter. (**B**) Representative confocal micrograph of Baf-pretreated cells fixed after 3 hpi and immunostained for major viral capsid protein p72 (red) and LE marker Rab7 (green); Bar 10 µm. Detail of colocalization between viral capsids and LEs in Baf-treated cells; Insets are magnifications of the boxed areas in the previous image, bar 1 µm. (**C**) Quantification of colocalization events relativized to the total number of cell-associated virions per individual cell, performed in 130 virions and expressed as means and standard deviations from two independent experiments. Asterisks denote statistically significant differences (***P<0.001).

In order to address the temporal relevance of low endosomal pH, we treated cells with NH_4_Cl at 10 mM concentration or 200 nM Baf to inhibit endosome acidification at several post-infection times. Blockage of acidification within the first hour of infection had a strong negative impact on early viral protein synthesis, as shown by p30 expression using both drug inhibitors (Fig. 3 A). Conversely, when added at later times (3 hpi), both Baf- and NH_4_Cl-treated cells produced p30 levels similar to control cells. Taken together, these results suggest that luminal endosomal acidification is a major determinant for allowing the virus to gain entry to the cytosol. LE passage would be required for ASFV uncoating and the low pH in this endosomal compartment resulted necessary within the first hour of infection when ASFV desencapsidation and viral egress take place.

The next question was whether endosomal acidification inhibition resulted in a demonstrable observation of viral capsid protein p72 in LEs, as would be expected from the inhibition of virus desencapsidation and release from this compartment. For this purpose, we examined the association of viral particles with LE marker Rab7 in these inhibitory acidic conditions. To this end, cells were treated for 20 min with 200 nM of Baf and then infected at 10 pfu/cell, after an adsorption period of 90 min at 4°C. Medium was then replaced with fresh medium containing Baf at 37°C, and these cells were compared with untreated infected cells in the same conditions. Infections were allowed to progress for 3 h in the presence of this agent. Cells were then fixed, immunolabeled with anti-Rab7 and anti-p72 and then analyzed using confocal microscopy.

Colocalization events between p72 and Rab7 increased significantly under inhibited acidification as shown at a higher magnification of Rab7-positive vesicles and associated viral particles (Fig. 3 B). Percentages of virions colocalizing with Rab7 per cell in control and in Baf-treated conditions are shown in graphics (Fig. 3 C). Increased percentages of p72 colocalization events with Rab7 when acidification was inhibited, strongly suggest that virion desencapsidation was blocked in a Rab7 staining LE compartment. Taken together, these observations are consistent with the notion that ASFV uses the endosomal pathway for infection and that an increase in the pH of acidic organelles can cause infection blockage, as shown by the retention of encapsidated viral particles in LEs in Baf-treated cells. These results would support that virus uncoating occurs through a transient step from the LE compartment.

These observations pointed to a crucial role of the LE compartment during the viral uncoating and egress from the endosome.

### Relevance of the late endosome compartment in ASFV infection

The relevance of the LE compartment in infection was further evaluated on infectivity and viral protein expression by transient expression of Rab7 dominant negative (DN) mutant (GFP-Rab7 DN; T22N) in COS-7 cells 24 h after transfection. This cell line was used in this experiment because it shows higher transfection efficiency than Vero cells.

**Figure 4:**
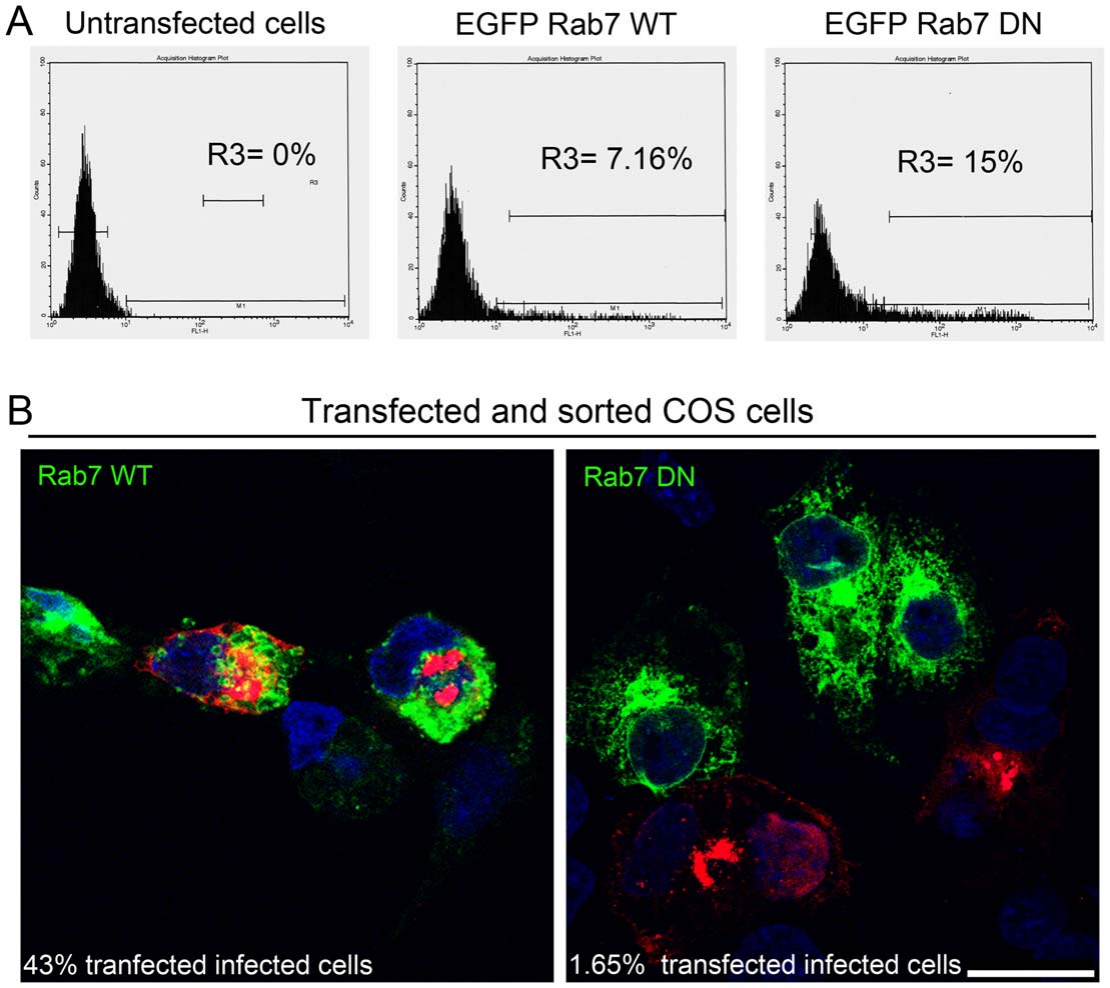
Late endosomal compartment relevance for ASFV infection. (**A**) Representative FACS profiles obtained during sorter analysis of COS-7 cells transfected with GFP-Rab7-wild type (Rab7 WT) and dominant negative mutant (GFP-Rab7-DN, T22N). R3 represents transfected cells expressing GFP to be sorted. (**B**) Representative confocal micrographs of transfected, sorted cells after isolation, infected with ASFV at a moi of 1 for 24 hpi and immunostained for major viral capsid protein p72 (red). Percentages of transfected infected cells decreased from 43.5% in cells expressing Rab7 WT to 1.65% in cells expressing Rab7 DN. Bar 25 µm.

Fluorescence-expressing cells were then sorted and isolated for subsequent infection, which was allowed to progress to complete viral cycle at 24 hpi. At this time point infected cells show a characteristic viral factory at the MTOC where viral proteins and viral DNA accumulate for the assembly of newly formed virions. Fig. 4 A shows the percentages of sorted transfected cells. Indirect immunofluorescence assay revealed that, from total counts of 400 cells per experiment, the infected cell number detected with a mouse monoclonal anti-p72 decreased from 43.5% for Rab7 WT-expressing cells to 1.65% for cells transfected with Rab7 DN plasmid (Fig. 4 B left and right panel, respectively).

Indirect fluorescent immunoassays (IFI) demonstrated that the expression of Rab7 DN decreased infectivity. Taken together, these data indicate that ASFV requires functional LE trafficking for infection, thereby suggesting that this step is necessary for successful viral infection. We propose that the LE environment provides the correct pH for ASFV uncoating and virus egress from the endosome to the cytosol.

### ASFV entry depends on endosomal membrane phosphoinositides

PIs are required for the maturation of endosomes as they progress along the endosomal pathway. To further study the relevance of the endocytic pathway in viral traffic, we then analyzed the role of endosomal membrane PIs. There are two main PIs involved in the regulation of the endosomes, PtdIns3P and phosphatidylinositol 3, 5 bisphosphate (PtdIns(3,5)P_2_).

**Figure 5:**
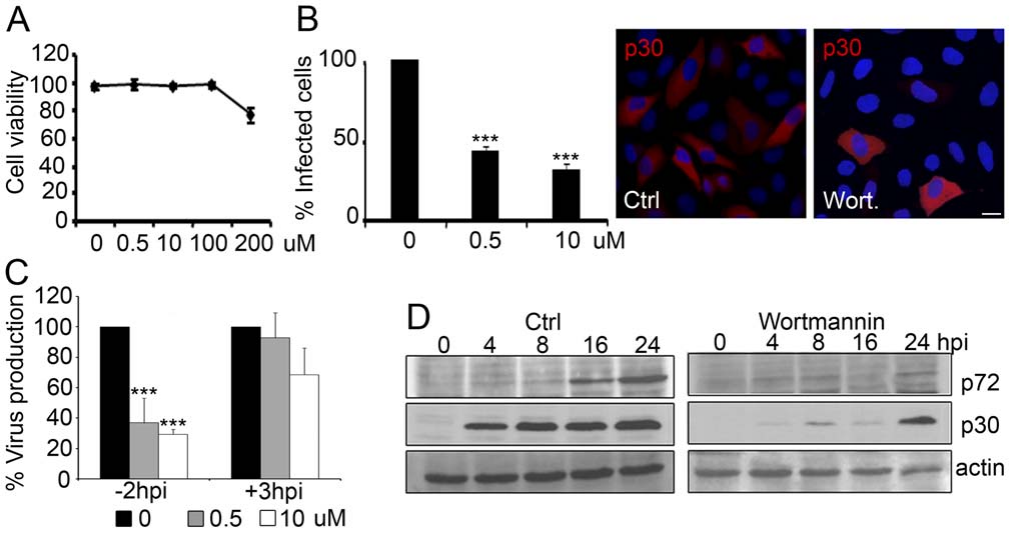
ASFV entry depends on endosomal membrane phosphoinositides. (**A**) Quantification of cell viability at 24 h by Trypan blue exclusion to determine the working concentration of PI3K inhibitor wortmannin. (**B**) Quantification of ASFV infectivity at 3 hpi (moi of 0.5 pfu/cell) in the presence of increasing concentrations of wortmannin. Data are expressed as percentages of infected cells from 30 random fields in triplicates and are means ± SD from three independent experiments. Asterisks denote statistically significant differences ***P<0.001. Representative confocal micrographs of cells immunostained for early viral protein p30 in red are shown in the right panels. Bar 20 µm. (**C**) Quantification of virus production in Vero cells untreated, treated with increasing concentrations of wortmannin from 2 h before adsorption during the whole infection cycle, or treated after 3 hpi. Cells were infected with ASFV at a moi of 0.5 pfu/cell for 24 hpi. Data are expressed as virus titers and are means ± SD from three independent experiments. Asterisks denote statistically significant differences ***P<0.001. (**D**) Viral protein expression at a range of post-infection times as determined by Western blot in cells to which 10 µM wortmannin was added 2 h before virus adsorption and maintained or left untreated.

**Figure 6:**
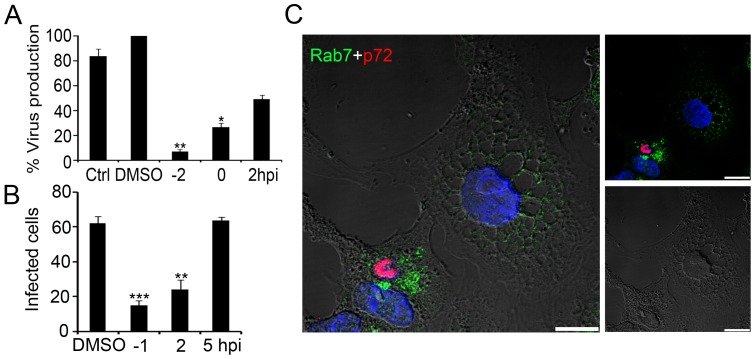
Phosphoinositide interconversion and related late endosome fusion events in ASFV infection. (**A**) Quantification of virus production in cells untreated, treated with 1 µM PIKfyve inhibitor YM201636 or treated with DMSO. Data are expressed as virus titers and are means ± SD from three independent experiments. Asterisks denote statistically significant differences **P<0.01; *P<0.05 (**B**) Infected cell numbers in cells treated with PIKfyve inhibitor (YM201636) at several time points or an equivalent volume of DMSO. Data are expressed as the number of infected cells at 6 hpi (moi of 1 pfu/cell) from 20 random fields and are means ± SD from two independent experiments. Asterisks denote statistically significant differences (***P<0.001 and **P<0.01). (**C**) Representative confocal micrographs of infected and non-infected PIKfyve-treated cells, immunostained for Rab7 (green) and viral protein p72 (red). The characteristic phenotype of cytoplasmic vacuoles due to impaired endosome fusion was readily found in uninfected cells. Infected cells are recognized in the image as those harboring viral factories in red and lacked cytoplasmic vacuolization phenotype. Bar 10 µm.

PtdIns3P itself and most if not all PtdIns3P-binding proteins that have been characterized, are present on early endosomes. Examples of those are the Rab5 effectors such as EEA1. PtdIns3P is found in a complex with Rab5 and EEA1, and the latter bridges the complex by binding PtdIns3P directly through its FYVE domain and Rab5 through its Rab5-binding domain. PtdIns3P therefore plays a fundamental role in endosomal trafficking as the anchoring element of protein complexes. Then, we first addressed the relevance of phosphoinositide 3 kinase (PI3K) in ASFV infection with the inhibitor drug wortmannin. Non-toxic concentrations were determined and low working concentrations (ranging from 0–10 µM) were used to avoid undesirable effects (Fig. 5 A). The addition of this inhibitor had a significant negative effect on infectivity in a dose-dependent manner, as shown by percentages of infected cells detected by p30 expression at 3 hpi, which corresponds to an early time point after virus entry but before replication (Fig. 5 B). The presence of low doses of wortmannin during the complete infection cycle reduced virus production in a dose-dependent manner; conversely, few changes were found when the drug was added after 3 hpi (Fig. 5 C). Viral protein production also decreased dramatically with the PI3K inhibitor (Fig. 5 D). Moreover, PtdIns(3,5)P_2_ is essential in LE/LY dynamics, with a distinct function from that of the small GTPase Rab7 [29], [30]. YM201636 is a potent inhibitor of the mammalian class III phosphatidylinositol phosphate kinase (PIKfyve), which synthesizes PtdIns(3,5)P_2_ from PtdIns3P. Then, we further evaluated the relevance of the correct maintenance of balance in the degradative pathway by analyzing the role of PIKfyve in ASFV infection using the inhibitor of this kinase. A working concentration of 1 µM of PIKfyve inhibitor was selected, this amount being non-toxic but active, as the characteristic swollen vesicle phenotype could be readily identified in cells (Fig. 6 C).

We studied whether the inhibition of PIKfyve activity before infection interferes with virus production. Cells were treated with 1 µM of YM201636 or equivalent volumes of DMSO at indicated times: 2 h prior to infection, at the time of infection (time 0) and at 2 hpi. The medium was then replaced with medium containing the drug at the indicated concentrations. Cells were infected at a moi of 1 pfu/cell and infection was allowed to proceed for 24 h. Statistically significant reductions in ASFV infectivity after YM201636 treatment correlated with a time-dependent decrease in viral production, with a maximum reduction of 90% in viral production in cells treated 2 h before infection with respect to DMSO control (Fig. 6 A).

We also examined the effect of PIKfyve inhibition on viral infectivity. To this end, 1 µM of YM201636 was added 1 h before or at several times after infection with ASFV at 1 pfu/cell. Controls without drug and equivalent volumes of DMSO were carried out. Infection was allowed to proceed for 6 h, an early time point that allows detecting early viral protein expression before viral replication occurs. IFI assays revealed a dramatic reduction of ASFV infectivity in cells treated with YM201636 from 1 h before infection and up to 4 hpi, but not thereafter (Fig. 6 B).

Due to the altered equilibrium between the respective PI levels towards PtdIns3P enrichment with respect to PtdIns(3,5)P_2_ after PIKfyve inhibition, there was a characteristic alteration in fusion dynamics and impaired maturation of endosomes. Morphologically, this alteration was characterized by the widespread appearance of large swollen vacuoles in most cytoplasmic areas of YM201636-treated cells (Fig. 6 C). YM201636-treated and infected cells at 16 hpi did not show this vacuolar pattern in 100% of those cells which were infected, as shown by a recognizable viral replication site that retained its characteristic morphology and location. This observation contrasted with the typical vacuolar pattern found in neighboring uninfected cells and the origin of this difference is not known (Fig. 6C).

## Discussion

ASFV enters Vero cells by endocytosis, through a dynamin-dependent and clathrin-mediated process [11]. However, the subsequent early steps followed by incoming virions to reach the virus replication site close to the MTOC are largely unknown. To identify the endosomal compartment/s involved in early steps of ASFV infection, we first searched for characteristic proteins of the EE, namely EEA1, and LE compartments; CD63 for MBVs, GTPase Rab7 for LEs and Lamp1 for LYs. EEA1 is a Rab5 GTPase effector that regulates the traffic and fusion events of EEs [21] while Rab7 GTPase controls the transport and fusion of LEs. ASFV virions stained with an antibody against major capsid protein p72 colocalized at high percentages with EEs within the first 30 min of infection, while colocalization with CD63, Rab7 and Lamp1 was very low at this time point. As the endosome associated with the incoming virus matures, the colocalization of viral particles with LEs would be expected; however, this colocalization was absent. We postulated that the virus desencapsidation occurs very rapidly in acidic endosomes and consequently it was difficult to observe colocalization of virions with LEs. This hypothesis is consistent with previous electron microscopy observations of viral cores in the cytoplasm of ASFV infected cells 60 min after adsorption corresponding to viral particles without their capsid, although it was not possible to observe the uncoating process itself [31]. In fact, using an antibody against a viral inner core protein (p150), it was possible to observe colocalization of viral cores with LEs while major viral capsid protein was not detected in this acidic late compartment.

We have shown here that endosomal acidification was a major determinant for allowing the virus to gain entry to the cytosol and pursue a productive infection. Inhibitors of intraluminal acidification of endocytic organelles were found to inhibit infection at any time point previous to 1 hpi but not thereafter. Endosomal intraluminal low pH was an important switch for incoming virions to progress into subsequent infection steps, as impaired ASFV infectivity by Baf could not be restored with acid pH treatment of cells. Moreover, the requirement for endocytosis was reinforced by the fact that dynamin inhibitor Dyn mediated infectivity inhibition and it was not possible to bypass this blockage by extracellular acid medium replacement. Similarly, it was previously reported that fusion of the cellular membrane artificially induced by lowering the pH of the medium [9] was not followed by successful ASFV infection in cells treated with other lysosomotropic drugs [10].

We found staining of viral capsid in LEs at intraluminal alkaline conditions, which confirmed the requirement of acidic pH, distinctive of the LE, for viral desencapsidation and further endosomal egress, which take place within the first hour post-infection in this virus model. This observation is consistent with previous results on the accessibility of viral DNA to nuclease and the timing of early viral RNA and protein synthesis of ASFV [31]. Similarly, a recent report on Simian virus 40 (SV40) showed that elevation of vacuolar pH blocks SV40 infection using acidification inhibitors Baf and NH_4_Cl [32]. SV40 intracellular traffic includes passage through EEs, maturing hybrid endosomes, LEs with the properties of MVBs, and finally endolysosomes. Agents that raise the pH of endocytic organelles were found to inhibit infection and the internalized virus fraction (about 20%) failed to move beyond Rab5-positive EEs in the presence of Baf and beyond LEs in the presence of monensin.

Under Baf-treatment desencapsidation would be stopped in Rab7-positive late endosomes, thus blocking viral infection progression. After reaching LE acid pH and uncoating would eventually egress from the endosome and the viral cores could be free in the cytosol to start replication at the perinuclear area. There is a close relationship between endosomal maturation and movement. One of the steps required for endosomal maturation includes endosome progression towards the perinuclear area [33], which is achieved through microtubules [30], [34]. Endosomal trafficking of ASFV relies on microtubules and previous reports have shown that this virus requires functional microtubules for successful infection [35]. Furthermore, Rac1 activation, which triggers microtubule acetylation and stabilization, is crucial for infection at early time points [36].

The specific low pH of the LE is required for many virus infections, such as the influenza A virus [32] and bunyavirus [37]. Also, most adenovirus (Ad) serotypes enter cells by clathrin-mediated endocytosis, and then the pH inside the endosomes plays an essential role by inducing conformational changes in the viral particle. Ad5 exposed to acidic pH levels shows a clear enhancement in dynein binding through intermediate and light intermediate chains. These data provide physiological evidence of the relevance of adenovirus exposure to endosomal pH for efficient infection [38]. Similarly, rhinovirus enters the cell via clathrin-dependent or -independent endocytosis or via macropinocytosis. Triggered by the low pH of endosomes, the virions undergo conformational alterations and the viral RNA genome is then released through an opening at one of the axes of the icosahedral capsid [39]. Also, Dengue virus uses the unusual lipid composition of the LE membrane for low pH-dependent virus fusion, which determines the timing and site of viral genome release into the cytosol [40].

We have demonstrated that Rab7 GTPase from the LE compartment is essential for successful infection. Rab7 GTPase is characteristic of LEs since the formation of this compartment is preceded by the generation of Rab7 domain, but this protein is scarce on the limiting membrane of EEs [33]. Transient expression of Rab7 DN severely affected ASFV infection outcome, as occurs with other enveloped viruses, such as the influenza virus [41]. After infection of sorted Rab7 WT and DN-transfected cells, ASFV infectivity decreased dramatically compared to cells expressing Rab7 WT.

Altering the balance in PI interconversion would disrupt the endocytic pathway. The small GTPase Rab5 and PtdIns3P are present on classical EEs where they coordinate the assembly of crucial effector complexes for the function and further maturation of these organelles. Efficient recruitment of some of these effectors, such as EEA1 between others, is based on their simultaneous binding to Rab5 and PtdIns3P [23]. As shown above, ASFV requires maturation of the EE to LE, and this process is upregulated by PI3K since the component of switch interconversion Rab5-Rab7, Sand1/Mon1, requires PtdIns3P for endosome binding [42]. Also, the formation of a functional MVB requires the biosynthesis of the membrane lipid PtdIns3P by PI3K [43]. The inhibition of PtdIns3P synthesis impaired ASFV infection, as expected, but not when it was inhibited after the early internalization steps. Thus, PtdIns3P concentration on endosomes regulates the timing of Rab conversion and endosome maturation, which directly affect the very early stages of ASFV infection before endosomal egress. Other viruses such as Kaposi-sarcoma, are also affected by wortmannin treatment [44]. Similar observations have recently been reported for parvoviruses [45], human rhinovirus serotype 2 (HRV2) [46], Influenza A and bunyavirus, all of these late-penetrating viruses [37].

Moreover, PtdIns3P is a precursor for the generation of PtdIns(3,5)P_2_ and is distinct from that of the small GTPase Rab7 [47] as it binds the FYVE domain containing PIKfyve [29]. The PIKfyve inhibitor YM201636 affects conversion from PtdIns3P to PtdIns(3,5)P_2_ [48], thereby triggering disruption of the degradative pathway and imbalance of fusion endosome dynamics [49], resulting in endosome enlargement and profound vacuolation in mammalian cells [19], [49], [50], [51]. We found that both PIKfyve and acidification inhibition had a strong negative impact on ASFV infection. This inhibitor decreased infectivity and viral production when YM201636 was added before infection but not after 2 hpi. These results with PIKfyve inhibitor are consistent with ASFV requirements for pH acidification during early infection stages since efflux of cations such as Ca^2^+ affects acidification [30], [52]. PIKfyve may directly regulate the activity of calcium channels and enable the efflux of Ca^2^+ allowing the regulation of membrane trafficking pathways in a spatiotemporal manner [53], [54].

In conclusion, the profound alteration of the maturation of the LE compartment caused by PIKfyve inhibition deeply affects ASFV infection, as occurs with other pathogens which are dependent on the LE compartment [55]. Conversely, after infection, when the stage of viral replication site formation is reached, it was not possible to further inhibit LE maturation by PIKfyve inhibition.

This is the first report on the requirement for endosomal maturation up to LEs for early ASFV infection. Our results have been achieved using several approaches, namely by blocking pH acidification, impairing the function of the regulatory GTPase of the LE compartment Rab7, and finally by impairing the synthesis of regulatory PIs that have an indisputable role in endosomal maturation.

**Figure 7:**
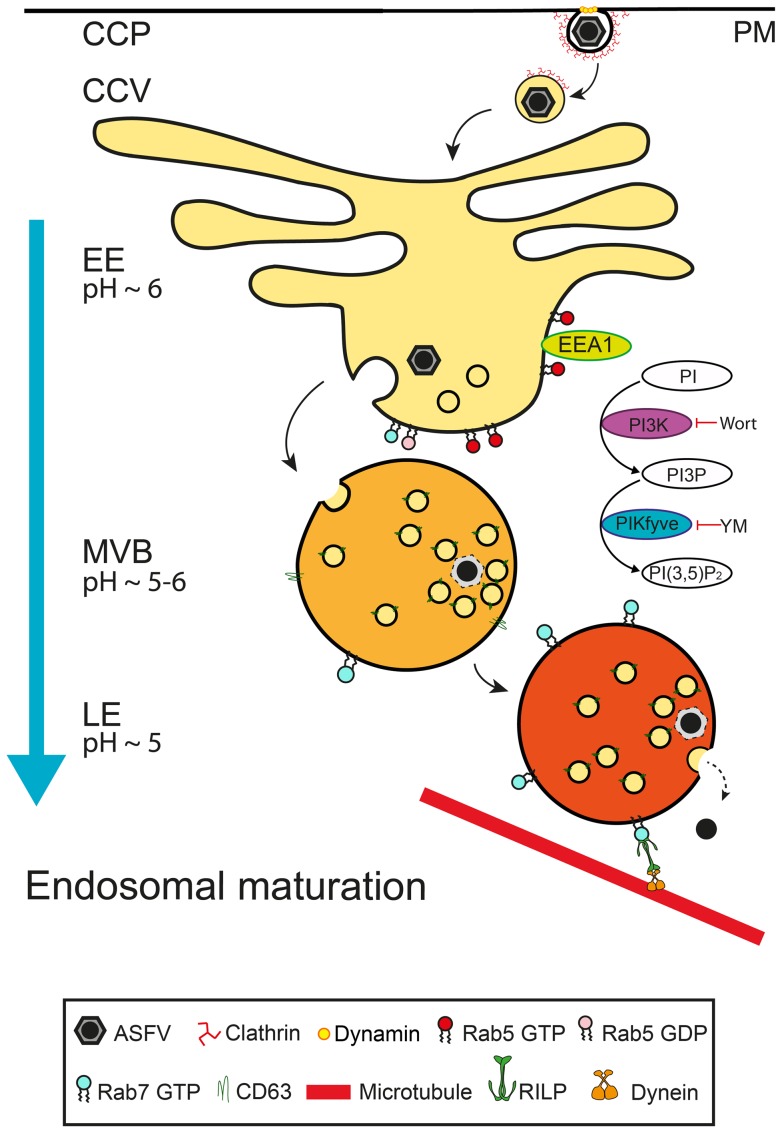
Model of ASFV infection progress through the endosomal pathway. ASFV enters the host cell by clathrin-coated vesicles (CCVs) from clathrin-coated pits (CCPs) and clathrin molecules are recycled to the plasma membrane (PM) as the virus progresses to the endosomal pathway. First, virions gain access to EEs from PM. The EE compartment is characterized by the presence of Rab5 and EEA1. ASFV is then directed from the vacuolar domain of the EE to the acidic late compartments. Subsequently, the virions reach CD63 enriched membranes of MVBs. Under the acid intraluminal pH of these endosomes, viral capsid would be degraded and viral cores would reach LE which depends on the presence of Rab7. At this stage, viral cores could egress to the cytosol to reach their replication site at the perinuclear area. In this process, the PIs composition of the endosomal membrane seemed to be crucial. PtdIns3P is synthesized by PI3K and this process is inhibited by PI3K inhibitor wortmannin and PtdIns(3,5)P_2_, which is synthesized by the enzyme PIKfyve, a process blocked by the inhibitor YM201636. These PIs interconversions on the endosomal membrane are necessary for a successful infection.

Taken together, our findings support a model where both EEs and LEs are required for successful ASFV infection (Fig. 7). The incoming virus would gain access to the EE immediately after entering the cell from the clathrin-coated vesicle. Upon EE maturation, the virus would be incorporated into MVBs, progressing to reach the LE and due to the acidic pH of these organelles, desencapsidation would take place. This step was found to be essential for further infection progress. This proposed model includes central roles for small GTPase Rab7 and also membrane PIs, PtdIns3P and PtdIns(3,5)P_2_, which orchestrate the maintenance of homeostasis along the pathway. In conclusion, we propose that the integrity of the endocytic pathway maturation process mediated by PIs play a central role during early stages of ASFV infection.

## Materials and Methods

### Cells, virus and infections

Vero and COS-7 cells were obtained from ATCC and grown at 37°C in a 5% CO2 atmosphere in Dulbecco's Modified Eagle's Medium (DMEM) supplemented with 5% and 10% fetal bovine serum (FBS), respectively. Cells were grown on chamber slides (Lab-Tek; Nunc), approximately 1.5×10^4^ cells/chamber and mock-infected or infected with ASFV-BA71V isolate or recombinant ASFV B54GFP-2 [56] at a multiplicity of infection (moi) of 1 or 10 pfu/ml when indicated. High moi was used to visualize several incoming virus particles/cell.

ASFV stocks from culture supernatants were clarified and semi-purified from vesicles by ultracentrifugation at 40,000 *g* through a 40% (wt/vol) sucrose cushion in phosphate-buffered saline (PBS) for 1 h at 4°C. Purified ASFV stocks were sonicated on ice once for 1 min and stored at −80°C. When synchronization of infection was required, cells were chilled at 4°C for 15 min before viral inoculum addition and virus was then added. Virus adsorption was performed for 90 min at 4°C, and after cold washing, cells were rapidly shifted to 37°C with fresh pre-warmed media.

### Indirect immunofluorescence and confocal microscopy

Cells were grown on glass coverslips and fixed in PBS–3.8% paraformaldehyde for 15 min and permeabilized with PBS–0.1% Triton X-100 for 10 min. Following cell fixation, aldehyde fluorescence was quenched by incubation of cells with 50 mM NH4Cl in PBS for 10 min. A monoclonal antibody against major virus capsid protein p72 and against viral core protein p150 (Ingenasa) were used at a working dilution of 1∶1000, an anti-p30 antibody at 1∶100 [57] and a rabbit serum raised against ASFV structural capsid protein pE120R at 1∶500 dilution. Experiments conducted to detect viral capsids were performed with antibodies against both capsid proteins p72 and pE120R and to detect viral cores the antibody against p150 was used. EE were labeled with conjugated anti-mouse EEA1-FITC (BD Biosciences Pharmingen), EEA1 being a Rab5 GTPase effector, and anti-rabbit Rab7 (Cell Signalling) was used to label LEs both at 1∶50 dilution. MVBs were labeled with anti CD63 (Developmental Studies Hybridoma Bank, University of Iowa, clone H5C6), a characteristic protein of this compartment, at 1∶200 dilution. LYs were labeled with anti-Lamp1 (Abcam) at 1∶50 dilution. The secondary antibodies used were anti-mouse immunoglobulin G (IgG) antibody conjugated to Alexa Fluor 594 and anti-rabbit IgG antibody conjugated to Alexa Fluor 488. Secondary antibodies were purchased from Molecular Probes and diluted 1∶200. Specificity of labeling and absence of signal crossover were determined by examination of single labeled control samples.

Confocal microscopy was carried out in a Leica TCS SPE confocal microscope using a 63X immersion oil objective, and image analyses were performed with Leica Application Suite advanced fluorescence software (LAS AF). Colocalization events between viral and endosome markers in each cell were counted and relativized to the number of total cell-associated virions and expressed in percentages. Graphics in figures depict means of this percentages and the number of cells counted in each case are expressed in figure legends.

### Lysotracker assays

Acid endosomal compartments were labeled by incubation of cells with 75 nM LysoTracker Red DND-99 (Molecular Probes) for 30 min at 37°, then Vero cells were prepared for IFI assay, double labeling endosomal compartments as above described.

To analyze the effect of Baf on LysoTracker staining, pretreated cells were incubated with Baf 200 nM and *in vivo* imaged at several times after Baf addition (1, 5, 10, 15 mpa or minutes after Baf addition), or IFI assays were performed. Fluorescence Intensity in arbitrary units was measured with LAS AF in three maximum points of fluorescence (three similar ROIs, 30×30 µm) per image and background was subtracted. 32 images per condition were analyzed.

### Inhibition of endosomal acidification

Stock solution of Baf (Sigma) was dissolved in DMSO at 100uM and stored at −20°C. A working concentration of 200 nM Baf [58], [59] was prepared freshly in DMEM. Stock solution of NH4Cl (Sigma) was made in PBS 1 M and the 10 mM working solution [10] was prepared freshly in DMEM. Stock solution of Dyn (Calbiochem) was prepared at 10 mM in DMSO. Cells were seeded at 70% confluence. The culture medium was replaced by cold medium and cells were placed at 4°C during 15 min. ASFV (moi of 1 pfu/ml) was then added on cold medium and the adsorption step was followed for 90 min at 4°C. After cold washing, cells were rapidly shifted to 37°. At the different times post-infection analyzed, culture medium was replaced by prewarmed medium containing Baf or NH4Cl or DMSO solvent. At 8 hpi, cells were harvested for SDS-PAGE analysis.

For the acid-pH treatment experiments, cells were pretreated with 200 nM Baf, 80 μM Dyn or solvent DMSO. Following, cells were rapidly chilled to 4°C before the addition of the virus. After 90 min adsorption at 4°C, cells were washed and pulsed with pH 5.4 DMEM for 1 h followed by washing and incubation for 6 h at 37°C with pH 7.4 DMEM.

For IFI assays, cells were untreated or pretreated with cold medium containing either Baf or an equivalent volume of DMSO for 20 min. Virus was then added at a moi over 10, followed by an adsorption step of 90 min at 4°C. After washing, culture medium was replaced with fresh pre-warmed medium with or without Baf or an equivalent volume of DMSO. Infection was allowed to progress for 3 h and prepared for IFI assay.

### Flow Cytometry

Cells were pretreated for 1 h with 80 µM Dyn or 200 nM Baf followed by cold synchronized infections with 1 pfu/ml ASFV and then washed with ice-cold DMEM to remove unattached virus before incubation with either an hour pulse of pH 5.4 DMEM followed by washing or pH 7.4 DMEM in the presence of inhibitor for the duration of the experiment. At 6 hpi cells were washed with PBS and harvested by trypsinization. After washing with fluorescence-activated cell sorter (FACS) buffer (PBS, 0.01% sodium azide, and 0.1% bovine serum albumin [BSA]), cells were fixed and permeabilized with Perm2 (BD Sciences) for 10 min at room temperature. Detection of infected cells was performed by incubation with anti-p30 monoclonal antibody (diluted 1∶100 in FACS buffer) for 30 min at 4°C, followed by incubation with phycoerythrin (PE)-conjugated antimouse immunoglobulins (1∶50, diluted in FACS buffer [Dako]) for 30 min at 4°C. After extensive washing, 10000 cells per time point were scored and analyzed in a FACSCalibur flow cytometer (BD Sciences) to determine the percentage of infected cells under these conditions. Infection rates obtained were normalized to infected cell percentages found in control plates.

### Viral protein expression analysis

Infected Vero cells were harvested at various times, washed in PBS, and disrupted in Laemmli sample buffer reducing agent (Bio-Rad) containing lithium dodecyl sulfate sample buffer. Lysates were sonicated and incubated at 100°C for 5 min and resolved by sodium dodecyl sulfate-polyacrylamide gel electrophoresis (SDS-PAGE) in a 10% gel. Proteins were transferred to a nitrocellulose membrane and blocked with PBS supplemented with 5% non-fat dried milk for 1 h at room temperature or overnight (ON) at 4°C. To detect p30 protein and α-tubulin, the latter used as a protein load control, membranes were incubated with an anti-p30 monoclonal antibody diluted 1∶1000 as previously described [57] and α-tubulin antibody diluted 1∶2000. As secondary antibody we used horseradish peroxidase (HRP)-coupled anti-mouse antibodies diluted 1∶5000 (GE Healthcare). Precision Protein StrepTactin-HRP Conjugate (Bio-Rad) was used to reveal the ladder Precision Plus Protein WesternC (Bio-Rad).

Western blots were analyzed using Immun-Star WesternC Kit (Bio-Rad) on Molecular Imager Chemidoc XRSplus Imaging System. Bands were quantified by densitometry and data were normalized to control values using Image lab software (Bio-Rad).

### Transfections and sorter analysis

Wild-type GFP-tagged human Rab7 and dominant-negative mutant (Rab7 T22N) plasmid constructs cloned as N-terminal GFP fusions in the pGreenLantern vector (Gibco-BRL, Grand Island NY, USA) were kindly provided by Dr, Craig Roy, Yale University, USA. Transfections were performed by using the Fugene HD Transfection Reagent from Roche and following the manufacturer's recommendations. Briefly, COS cells were grown in T-75 flasks, in DMEM 10% serum with 1% streptomycin, penicillin and 1% glutamine until 80% confluence was reached and they were then transfected. After 6 h, the transfection mixture was removed and fresh medium containing 10% serum and 1% antibiotics and glutamine was added. Transfection was allowed to progress for 24 h. After 24 h post-transfection (hpt), cells were treated with trypsin for 1 min. Trypsin was then carefully removed and detached transfected cells were resuspended in DMEM 5% with 100 µg/µL gentamycin.

1.2×10^8^ cells were analyzed for each transfected vector in a Coulter flow cytometer with an argon laser at 488 nm. Cells expressing EGFP fluorescence were counted and seeded into 4-well plates for 12 h. The next day, cells were infected with ASFV at a moi of 1 and analyzed by IFI assay.

### Phosphoinositide interconversion inhibitors

Wortmannin, an inhibitor of phosphoinositide 3 kinase (PI3K), which impairs PtdIns3P production, was purchased from Stressgen. The inhibitor did not affect cell viability, as tested by Trypan blue staining. Cells were treated with the drug following three protocols. First, drug treatment was maintained during the entire experiment (24 h). Second, the drug was added before infection and maintained along viral adsorption (cells were rinsed with fresh media after adsorption to remove the drug). Finally, in the third protocol we added the drug 3 h after viral adsorption and maintained it throughout the experiment.

PIKfyve Inhibitor YM201636, which impairs PtdIns(3,5)P_2_ production from PtdIns3P, was purchased from Symansis (Cell Signaling Science). A stock solution was diluted in DMSO at 0.8 mM concentration and stored at −20°. A cytotoxicity assay “Cell titter 96” from Promega was used to determine the working concentration of 1 µM as the lowest non-cytotoxic concentration inducing a characteristic swollen vesicle phenotype in the cytoplasm after 30 min incubation. Cells were treated with YM201636 2 hours before infection or at the times indicated and maintained during the whole infection cycle or at the post-infection times indicated in each case.

## Supporting Information

Figure S1 **Staining of the different endosomal compartments with pH sensitive dye.** (**A**) Absence of lysotracker red staining in the EE demonstrates the alkaline intraluminal pH of these organelles. (**B**) MVBs showed lower pH and lysotracker red labeling of these organelles is shown in yellow in the merged image. Similarly, LEs (**C**) and LYs (**D**) intraluminal acid pH is shown by lysotracker staining. Bar 10 µm.
(TIF)
