# Supplementary figures and images for: Endosomal Maturation, Rab7 GTPase and Phosphoinositides in African Swine Fever Virus Entry
Source: PLoS One. 2012 Nov 1;7(11):e48853. doi: 10.1371/journal.pone.0048853 (PMC3486801; doi:10.1371/journal.pone.0048853)

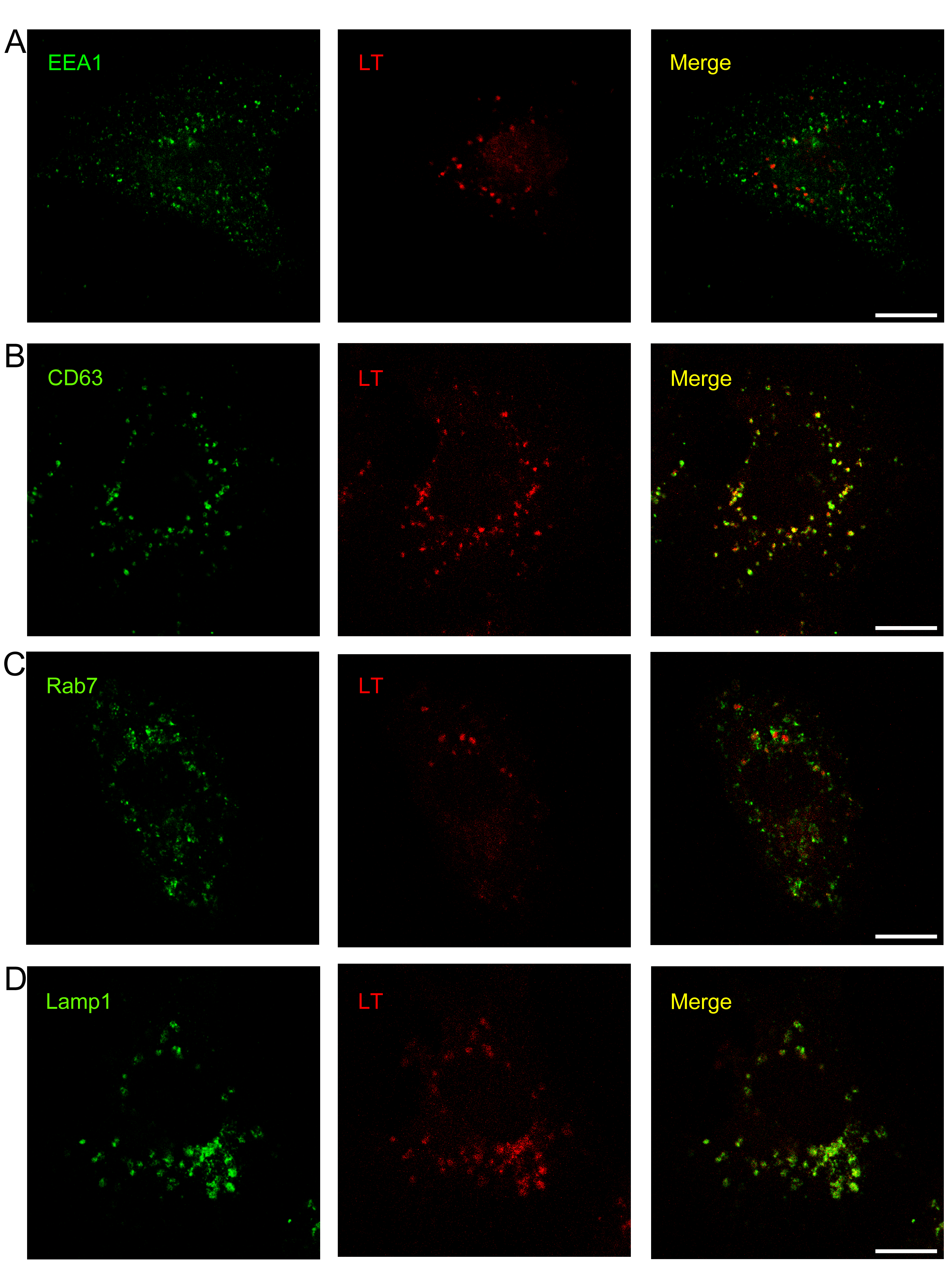

Supplement: Figure S1 — Staining of the different endosomal compartments with pH sensitive dye. (A) Absence of lysotracker red staining in the EE demonstrates the alkaline intraluminal pH of these organelles. (B) MVBs showed lower pH and lysotracker red labeling of these organelles is shown in yellow in the merged image. Similarly, LEs (C) and LYs (D) intraluminal acid pH is shown by lysotracker staining. Bar 10 µm. (TIF) [file pone.0048853.s001.tif]
